# Effects of escitalopram on synaptic density in the healthy human brain: a randomized controlled trial

**DOI:** 10.1038/s41380-023-02285-8

**Published:** 2023-10-09

**Authors:** Annette Johansen, Sophia Armand, Pontus Plavén-Sigray, Arafat Nasser, Brice Ozenne, Ida N. Petersen, Sune H. Keller, Jacob Madsen, Vincent Beliveau, Kirsten Møller, Alexandra Vassilieva, Christelle Langley, Claus Svarer, Dea S. Stenbæk, Barbara J. Sahakian, Gitte M. Knudsen

**Affiliations:** 1 Neurobiology Research Unit, Copenhagen University Hospital Rigshospitalet, Copenhagen, Denmark; 2 Department of Clinical Medicine, Faculty of Health and Medical Sciences, University of Copenhagen, Copenhagen, Denmark; 3 Department of Psychology, Faculty of Social Sciences, University of Copenhagen, Copenhagen, Denmark; 4 Department of Public Health, Section of Biostatistics, University of Copenhagen, Copenhagen, Denmark; 5 Department of Clinical Physiology and Nuclear Medicine, Copenhagen University Hospital Rigshospitalet, Copenhagen, Denmark; 6 Department of Neurology, Medical University of Innsbruck, Innsbruck, Austria; 7 Department of Neuroanaesthesiology, Copenhagen University Hospital Rigshospitalet, Copenhagen, Denmark; 8 Department of Psychiatry, University of Cambridge, Cambridge, UK

**Keywords:** Biomarkers, Psychiatric disorders, Neuroscience, Molecular biology

## Abstract

Selective serotonin reuptake inhibitors (SSRIs) are widely used for treating neuropsychiatric disorders. However, the exact mechanism of action and why effects can take several weeks to manifest is not clear. The hypothesis of neuroplasticity is supported by preclinical studies, but the evidence in humans is limited. Here, we investigate the effects of the SSRI escitalopram on presynaptic density as a proxy for synaptic plasticity. In a double-blind placebo-controlled study (NCT04239339), 32 healthy participants with no history of psychiatric or cognitive disorders were randomized to receive daily oral dosing of either 20 mg escitalopram (*n* = 17) or a placebo (*n* = 15). After an intervention period of 3–5 weeks, participants underwent a [^11^C]UCB-J PET scan (29 with full arterial input function) to quantify synaptic vesicle glycoprotein 2A (SV2A) density in the hippocampus and the neocortex. Whereas we find no statistically significant group difference in SV2A binding after an average of 29 (range: 24–38) days of intervention, our secondary analyses show a time-dependent effect of escitalopram on cerebral SV2A binding with positive associations between [^11^C]UCB-J binding and duration of escitalopram intervention. Our findings suggest that brain synaptic plasticity evolves over 3–5 weeks in healthy humans following daily intake of escitalopram. This is the first in vivo evidence to support the hypothesis of neuroplasticity as a mechanism of action for SSRIs in humans and it offers a plausible biological explanation for the delayed treatment response commonly observed in patients treated with SSRIs. While replication is warranted, these results have important implications for the design of future clinical studies investigating the neurobiological effects of SSRIs.

## Introduction

Drugs targeting the serotonin system, specifically the serotonin transporter, have long been the primary pharmacological treatment for affective and anxiety-related disorders [1]. The most widely used group is the selective serotonin reuptake inhibitors (SSRIs), presumed to work by increasing serotonergic neurotransmission [2]. Serotonin plays an important modulatory role in the brain, including regulation of mood, sleep, cognition, and behaviour, and in the early development of the central nervous system [3, 4]. Further, years of preclinical studies have established a link between the serotonin system and cellular processes such as cytoskeletal rearrangements, long-term potentiation, and neuronal firing – processes that collectively are regarded as forms of neuroplasticity [2, 5]. Functionally, neuroplasticity can be thought of as the ability of the brain to change and adapt to physiological or psychological stimuli to uphold homeostasis [6].

Despite years of research, the question of how inhibition of the serotonin transporter leads to symptom relief in neuropsychiatric conditions, remains unresolved. Major depressive disorder (MDD) is a vastly heterogeneous syndrome [7] and up to 35% of patients treated with SSRIs do not reach a state of remission [8]. Thus, a deeper understanding of the neurobiological effects of SSRIs, together with better patient stratification [9], is needed to tailor treatment to individual patients and pursue other treatment strategies for patients who are unlikely to benefit from SSRIs.

One hypothesis for the mechanism of action in neuropsychiatric disorders is that strengthened serotonergic neurotransmission induces neuroplasticity and, in turn, improves cognitive and emotion processing [10–12]. Neuroplastic effects have foremost been demonstrated for the visual system; in adult rats, chronic treatment with the SSRI fluoxetine has been shown to reactivate a critical period-like plasticity in the visual cortex [13, 14]. However, whether neuroplasticity is central to the effects of SSRIs in humans has been difficult to investigate, mainly due to the lack of specific biomarkers. A suggested proxy is a change in cortical thickness or brain volume, as measured with MRI, in response to, e.g., learning new skills or tasks, such as juggling [15]. However, by using PET, it is possible to non-invasively quantify molecular biomarkers that more specifically reflect plasticity in vivo. Here, we use the PET radioligand [^11^C]UCB-J that binds to the Synaptic Vesicle glycoprotein 2A (SV2A), which enables visualization and quantification of pre-synaptic density [16], as a proxy for synaptic plasticity.

PET studies on several neuropsychiatric disorders linked to synaptic dysfunction, including depression, have found lower cerebral SV2A density in patients compared to healthy individuals [17–22]. So far, the only investigation of a pharmacological intervention on SV2A density in humans is a study that examined the acute effect of a single administration of the rapid-acting antidepressant ketamine, and they found no changes for healthy participants and psychiatric patients 24 h after the intervention, but a post-hoc analysis indicated possible effects in a subgroup of patients [23]. Whereas ketamine’s psychoactive effects are hyper-acute, with antidepressant effects reaching a maximum one day after administration [24], the clinical effects of SSRIs emerge much slower. Some studies suggest that SSRIs have acute or subacute effects on cognition, e.g., affective processing bias [25–27], but it generally takes several weeks before symptom relief occurs in patients with depression [28–31]. This suggests that clinical effects result from neurobiological changes that emerge gradually, likely over the course of several weeks.

Given the limited knowledge of SSRIs’ neurobiological effects in humans, such as their capacity to induce neuroplasticity, we here aim to investigate if SSRI administration over several weeks can alter synaptic density in the healthy human brain, specifically in the hippocampus and the neocortex. The hippocampus is often the target of research on neuroplasticity as it is a key region in learning and memory, and patients with severe depression have been found to have lower SV2A in the hippocampus and several neocortical regions [20]. Although categorized as a *mood* disorder, symptoms of depression indicate global affection of the brain, with deficits related to, e.g., memory and executive function, that can improve independent of change in depression scores following SSRI treatment [32]. For this reason, we chose the global neocortex for our primary investigation rather than specific sub-regions.

Here, we used a double-blind, semi-randomized, placebo-controlled design to test the hypothesis that healthy participants receiving daily SSRI administration would have higher SV2A binding in the hippocampus and the neocortex than those receiving a placebo. We further hypothesized that SV2A binding would be positively associated with the duration of escitalopram intervention.

## Methods

### Study design

The study was conducted in conjunction with a cross-sectional (i.e., single-scan), double-blinded, semi-randomized, placebo-controlled study (see Supplementary Fig S2) on the cognitive effects of escitalopram [33] preregistered at ClinicalTrials.gov (NCT04239339). The study was conducted at the Copenhagen University Hospital, Rigshospitalet, between May 2020 and October 2021. Approval was granted by the Danish ethics committee for the capital region of Copenhagen (journal ID: H-18038352, with amendments 71579, 73632, and 78565).

All participants were recruited from a database of individuals who had expressed interest in participating in brain imaging studies. Following information about the study, including potential side effects of escitalopram, participants gave their written consent. Next, participants underwent a screening procedure, including medical history, physical and neurological examination, and screening for current or previous psychiatric disorders according to in- and exclusion criteria (see Supplementary file for complete list). Following the screening procedure and neuropsychological testing of IQ (assessed using the Reynolds Intellectual Screening Test (RIST) [34]) and reaction time, participants were semi-randomized to receive either escitalopram (20 mg daily in capsules of 10 mg) or a placebo in identical capsules that were manufactured and distributed by the Capital Region Pharmacy. The dose of escitalopram was chosen to reflect typical clinical practice (i.e., 10–20 mg) for treating conditions such as MDD, and to minimize the risk of a false-negative result due to low dosing.

Randomization balanced with regards to age, sex, and IQ was done by a research administrator not otherwise involved in data collection or analysis. Participants were instructed to take one capsule daily by mouth for three days and then increase to two capsules daily (i.e., full dose). The aim was an intervention period of a minimum 3 weeks, and for logistical purposes and to allow room for unforeseen events (e.g., illness or technical issues), participants could continue the intervention for up to 5 weeks. After the intervention period, all participants came in for extensive neuropsychological testing and MRI examination. On intervention day 10 and the day of neuropsychological testing and MRI, a blood sample was collected to measure s-escitalopram steady-state levels as confirmation of drug adherence. Participants were instructed only to take their daily dose of medication after the blood sample had been drawn. S-escitalopram was measured with an ultra-performance liquid chromatography-tandem mass spectrometer (UPLC-MS/MS; Filadelfia Epilepsy Hospital, Dianalund, Denmark).

The main study included 66 healthy participants, for which we have reported the neuropsychological outcomes [33]. A subset of 32 participants underwent [^11^C]UCB-J PET scanning after the main study program was completed and while still double-blinded to the intervention. Participants were asked at the time of inclusion whether they, in addition to the described study program, agreed to undergo a PET scan. The sample size for the PET cohort (16 participants in each group) was calibrated to detect a 10% change (Cohen’s d ≅ 1) in [^11^C]UCB-J *V*_T_ in the hippocampus, at 80% power and a significance level of 0.05, based on data from Finnema et al. [35]. The data presented here are based on these 32 participants.

### MRI acquisition and preprocessing

All participants underwent MRI scans in a Siemens Magnetom Prisma 3 T scanner (Siemens AG, Erlangen, Germany) using a Siemens 32-channel head coil. Structural T1- and T2-weighted images were acquired (T1 protocol: Isotropic 0.9 × 0.9 × 0.9 mm^3^ resolution, repetition time = 2000 ms, echo time = 2.58 ms, inversion time = 972 ms, and flip angle = 8°; T2 protocol: Isotropic 0.9 × 0.9 × 0.9 mm^3^ resolution, repetition time = 3200 ms, echo time = 408 ms). Grey matter masks for PET processing were extracted from T1- and T2-weighted images using the multispectral segmentation routine in SPM12 (Functional Imaging Laboratory, the Wellcome Trust Centre for NeuroImaging, London, UK). Cortical thickness and hippocampal volume were derived from the T1-weigthed images using the standard anatomical processing stream (recon-all) from FreeSurfer (v. 7.2, https://surfer.nmr.mgh.harvard.edu/) [36], with manual refinement of the pial surface using the T2-weighted images.

### PET acquisition

Radiosynthesis of [^11^C]UCB-J was modified on the basis of Nabulsi et al. [37], as described in detail in the Supplementary file. All participants were scanned with a high-resolution research tomography (HRRT) PET scanner (CTI/Siemens, Knoxville, TN, USA). Following a six-min transmission scan, a 120 min PET scan was started at the time of intravenous [^11^C]UCB-J bolus injection (over ~20 sec). PET data were acquired in 3D list mode and reconstructed into 40 frames (8 × 15 s, 8 × 30 s, 4 × 60 s, 5 × 120 s, 10 × 300 s, 5 × 300 s) using a 3D OP-OSEM algorithm with modelling of the point-spread-function [38, 39], and attenuation corrected using the HRRT maximum a posteriori transmission reconstruction method (MAP-TR) [40]. Each image frame consisted of 207 planes of 256 × 256 voxels of 1.22 × 1.22 × 1.22 mm^3^.

### Arterial blood acquisition and analysis

For determination of the arterial input function, arterial blood samples were collected from a 20 G catheter which had been placed in the radial artery under local anesthesia. For the first 15 min of each scan, whole blood radioactivity was continuously measured (2-s intervals, flow = 8 mL/min) using an Allogg ABSS autosampler (Allogg Technology, Mariefred, Sweden). In addition, manual blood samples were drawn at 2.5, 5, 10, 25, 40, 60, 90, and 120 min for measuring radioactivity in blood and plasma using a gamma counter (Cobra II auto-gamma, Packard, Packard Instrument Company, Meriden, CT, USA) that was cross-calibrated to the PET scanner biweekly. Plasma was extracted after centrifugation of arterial blood at 2246xg for 7 min at 4 °C. To measure intact tracer and radiolabeled metabolites, plasma samples up until 90 min were analyzed using radio-HPLC (see the Supplementary file for full detail).

The plasma free fraction (*f*_P_) of [^11^C]UCB-J was determined by the equilibrium dialysis method as described in the Supplementary file.

### PET image processing

All PET images were motion corrected using the AIR software with the reconcile command (Automated Image Registration, v. 5.2.5, LONI, UCLA, http://air.bmap.ucla.edu/AIR5/). Tissue time-activity curves were extracted from automatically defined ROIs using the PVElab software (https://nru.dk/index.php/allcategories/category/30-software). The neocortex ROI was defined as a weighted average of the individual subregions (frontal, parietal, temporal, occipital and insular cortices). The PVElab pipeline used an unfiltered summation PET image that was automatically co-registered to the participant’s T1-weighted MR image using SPM12. Segmented T1- and T2-weighted MR images were then used to extract grey matter values from each ROI defined with a brain atlas, as previously described [41]. Co-registration and ROI placement were visually inspected for each subject; no manual correction was needed. No correction for partial volume effects was applied. The ROI for the centrum semiovale (white matter) was obtained from the PVElab region with the Müeller-Gartner partial volume correction method and was further eroded twice with a 3D erosion operator to minimize partial volume effects. The final volume had a mean (SD) of 7.45 (2.63) mL.

### Kinetic modeling

Kinetic modelling of [^11^C]UCB-J PET data was performed in R (v. 4.2.2, R Foundation, Vienna, Austria) using the *kinfitr* package (v. 0.6) [42]. Time-activity curves from all ROIs were fitted to the one-tissue compartment model (1TCM) using the subject’s metabolite-corrected arterial input function to estimate the total volume of distribution (*V*_T_), an index of SV2A binding. The fraction of blood volume (*v*_B_) was excluded from the model as it did not improve the model fits or change *V*_T_ estimates, which is in agreement with previous kinetic evaluations [35].

In addition, as a complementary analysis, time-activity curves from the hippocampus and neocortex were fitted to the simplified reference tissue model 2 (SRTM2) to estimate the non-displaceable binding potential (*BP*_ND_) using the white matter region centrum semiovale as a pseudo-reference region [43, 44]. The median k2 from 1TC modelling of centrum semiovale was used as a global k2’ (0.035 min^−1^).

### Statistical analyses

The distributions of demographic variables and PET scan parameters were visually compared between the groups and formally tested with a Welch two-sample *t*-test for continuous variables and Chi-squared tests for group sex ratios. Our primary hypotheses of higher [^11^C]UCB-J *V*_T_ in the hippocampus and the neocortex in the escitalopram group compared to the placebo group were tested using Welch two-sample *t*-tests. As a sensitivity analysis, we also conducted group comparisons using general linear models with randomization variables (age, sex, and IQ) as covariates. Improvement of model fits was assessed with a likelihood ratio test comparison of nested models.

As a secondary analysis, we investigated if there was an effect on [^11^C]UCB-J *V*_T_ dependent on escitalopram intervention duration: using a likelihood-ratio test, we compared a linear regression model including a *group-by-intervention duration* interaction term to a nested model where the group term was excluded. The models were also performed with age, sex, and IQ as covariates. Partial correlation coefficients (*r*_ρ_) were calculated based on the linear models [45]. We further investigated the effect of s-escitalopram concentration (log-transformed) on [^11^C]UCB-J *V*_T_ using linear regression.

Group means for [^11^C]UCB-J *V*_T_ estimates for other regions are listed in the Supplementary file (Table S1). These include neocortical ROIs: Orbital frontal, anterior cingulate, insula, superior temporal gyrus, parietal, medial inferior temporal gyrus, superior frontal, occipital, sensory-motor, dorsolateral prefrontal gyrus, ventrolateral prefrontal gyrus. Subcortical ROIs: Centrum semiovale, thalamus, caudate, putamen, entorhinal cortex, amygdala, raphe nuclei. Neocortex and hippocampus *BP*_ND_s from the SRTM2 model were compared with two-sample t-tests.

As exploratory analyses, we investigated the effects of escitalopram versus placebo, intervention duration, and s-escitalopram concentration on hippocampus volume adjusted for age, sex, and intracranial volume (ICV). Lastly, for the neocortical subregions frontal, parietal, temporal, occipital, and insular cortex, we examined if there was a group and intervention duration effect on cortical thickness using linear regressions, as described for [^11^C]UCB-J *V*_T_s, with age and sex as covariates.

All tests were performed as two-sided tests. Secondary and exploratory analyses were corrected for multiple comparisons according to the number of regions investigated, using the Bonferroni-Holm method. Statistical analyses were performed in R (v. 4.2.2).

## Results

### Demographics and scan-related parameters

The escitalopram and placebo groups were similar in age, sex distribution, and PET-related variables, including [^11^C]UCB-J plasma free fraction (Table 1). This was also the case when leaving out three participants without full arterial input functions, all from the placebo group. Serum-escitalopram measurements confirmed the correct group assignment and that all participants in the escitalopram group had been compliant.

**Table 1:** Subject demographics and [^11^C]UCB-J PET scan-related parameters.

	Placebo (*N* = 15^1^)	Escitalopram (*N* = 17)	*p*-value
Sex			
Female	8 (53%)	12 (71%)	0.52
Male	7 (47%)	5 (29%)	
Age (years)			
Mean (SD)	22.8 (2.9)	25.2 (5.8)	0.15
Median [Min, Max]	21.7 [19.9, 31.6]	22.7 [19.6, 41.9]	
IQ			
Mean (SD)	108 (5.9)	112 (8.0)	0.11
Median [Min, Max]	108 [94, 118]	113 [99, 129]	
Intervention duration (days)			
Mean (SD)	30.4 (4.7)	28.2 (3.3)	0.14
Median [Min, Max]	32.0 [22.0, 38.0]	27.0 [24.0, 35.0]	
S-escitalopram, day 10 (nmol/L)			
Mean (SD)	0 (0)	86 (75)	-
Median [Min, Max]	0 [0, 0]	68 [28, 338]	
S-escitalopram, follow-up (nmol/L)			
Mean (SD)	0 (0)	84 (56)	-
Median [Min, Max]	0 [0, 0]	69 [28, 263]	
Injected dose (MBq)			
Mean (SD)	401 (101)	410 (63)	0.77
Median [Min, Max]	415 [124, 550]	414 [251, 526]	
Injected mass (ng/kg)			
Mean (SD)	12.2 (19.5)	8.9 (7.2)	0.53
Median [Min, Max]	8.8 [1.2, 80.9]	6.7 [1.4, 29.3]	
*f*_P_			
Mean (SD)	0.36 (0.05)	0.36 (0.05)	0.87
Median [Min, Max]	0.37 [0.29, 0.46]	0.38 [0.26, 0.42]	

*P*-values refer to two-sample* t*-tests for continuous variables and Chi-square tests for categorical variables.

^1^Includes three participants in the placebo group who did not have a complete arterial input function. Group characteristics (central tendency measures and spread) did not change noticeably when leaving out these participants.

### Primary analyses

**Fig. 1:**
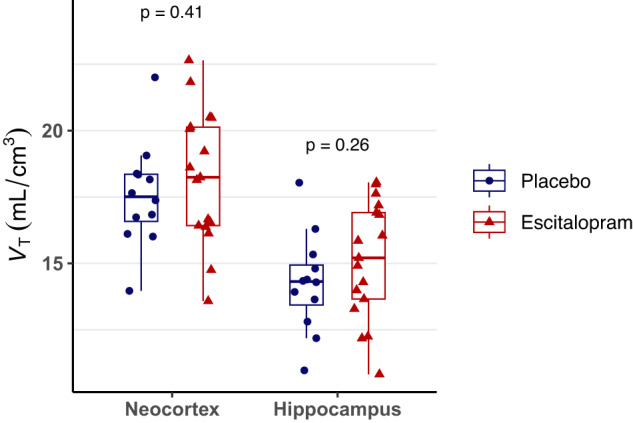
Effects of escitalopram on SV2A density. Comparison of [^11^C]UCB-J binding in healthy individuals following 3-5 weeks of intervention with escitalopram (*n* = 17) or placebo (*n* = 12). [^11^C]UCB-J total volume of distribution (*V*_T_) quantified using the 1TCM (*n* = 29).

There was no statistically significant difference in [^11^C]UCB-J binding between the escitalopram and placebo group in our primary ROIs, the hippocampus and the neocortex, after an average intervention period of 29 days (Fig. 1). The mean (SD) *V*_T_ in the hippocampus was 15.1 (2.2) mL/cm^3^ for escitalopram (*n* = 17) vs. 14.3 (1.9) mL/cm^3^ for placebo (*n* = 12), corresponding to Cohen’s *d* = 0.43 (95% CI [−0.36, 1.20], *p* = 0.26). In the neocortex, the mean (SD) *V*_T_ was 18.3 (2.5) mL/cm^3^ for escitalopram (*n* = 17) vs. 17.6 (2.0) mL/cm^3^ for placebo (*n* = 12) corresponding to Cohen’s *d* = 0.31, (95% CI [−0.47, 1.08], *p* = 0.41).

Including age, sex, and IQ as covariates did not reveal any significant group differences in the neocortex or the hippocampus (Supplementary Table S2). Likelihood ratios tests evaluating the improvement of model fits for the neocortex and the hippocampus resulted in the following p-values; *p* > 0.76 for age; *p* < 0.071 for sex; *p* < 0.014 for IQ. None of the covariates improved the model fit for the centrum semiovale (all *p*-values > 0.32).

Neocortical subregions and subcortical regions were not included in our a priori hypothesis; none of these regions showed significant differences in [^11^C]UCB-J *V*_T_ estimates between escitalopram and placebo groups as compared with Welch two-sample *t*-tests (Supplementary Table S1). For completeness, we also evaluated the non-displaceable binding potential (*BP*_ND_) based on reference tissue modelling using white matter as a reference region (Fig. S2). Mean (SD) *BP*_ND_ in the hippocampus was 2.65 (0.36) for escitalopram (*n* = 17) vs. 2.70 (0.38) for placebo (*n* = 15)(*p* = 0.67), while *BP*_ND_ in the neocortex was 3.42 (0.38) for escitalopram (*n* = 17) vs. 3.57 (0.42) for placebo (*p* = 0.31).

### Secondary analyses

#### Effect of intervention duration on [^11^C]UCB-J binding

**Fig. 2:**
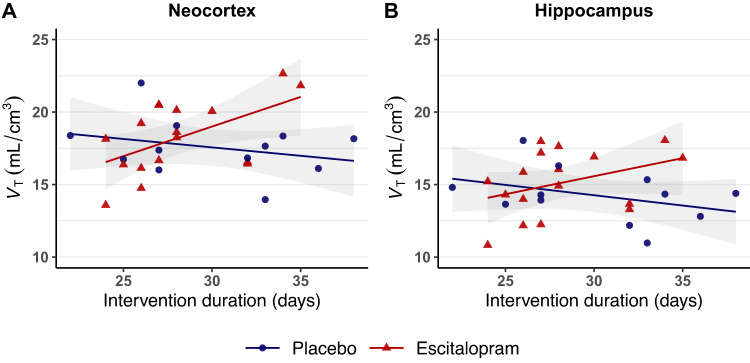
Time-dependent effects of escitalopram on SV2A density. Relationship between [^11^C]UCB-J binding (*V*_T_) and the duration of the intervention for the placebo group (*n* = 12) and the escitalopram group (*n* = 17) in the neocortex (**A**) and the hippocampus (**B**). The shaded grey area represents the 95% CI.

As the length of the intervention period ranged from 24 to 35 days for the escitalopram group, we investigated if longer exposure to escitalopram was associated with higher [^11^C]UCB-J *V*_T_. A likelihood-ratio test between a linear regression model including a *group-by-intervention duration* interaction term and a nested model where the *group* term was excluded, indicated a time-dependent group effect of escitalopram: the test resulted in a *p*-value of 0.020 (*p*_*adj*._ = 0.039) for the neocortex and 0.058 (*p*_*adj*._ = 0.058) for the hippocampus We then modelled the drug-specific effect of the duration of escitalopram intervention on [^11^C]UCB-J *V*_T_: in the neocortex (Fig. 2A) we found a positive effect of time for the escitalopram group, estimated to be +0.41 mL/cm^3^ per day (*r*_ρ_ = 0.46, *p* = 0.016), whereas it was −0.12 mL/cm^3^ per day (*r*_ρ_ = −0.18, *p* = 0.38) for the placebo group. Similarly, for the hippocampus (Fig. 2B), the effect of time on [^11^C]UCB-J *V*_T_ was +0.25 mL/cm^3^ per day (*r*_ρ_ = 0.31, *p* = 0.11) for the escitalopram group, whereas for the placebo group, it was −0.14 mL/cm^3^ per day (*r*_ρ_ = −0.22, *p* = 0.26).

When including age, sex, and IQ in the models, the effects of intervention duration in the escitalopram group were further strengthened: in the neocortex, the effect of intervention duration on [^11^C]UCB-J *V*_T_ was +0.47 mL/cm^3^ per day (*r*_ρ_ = 0.58, *p* = 0.003) for the escitalopram group, while there was no effect for the placebo group: −0.01 mL/cm^3^ per day (*r*_ρ_ = -0.01, *p* = 0.95). For the hippocampus, the effect of intervention duration was +0.30 mL/cm^3^ per day (*r*_ρ_ = 0.40, *p* = 0.048) for the escitalopram group, while there was no effect for the placebo group: −0.06 mL/cm^3^ per day (*r*_ρ_ = −0.11, *p* = 0.62). The effect of intervention duration was also observed for the centrum semiovale. All model estimates are listed in Supplementary Table S3.

#### Effect s-escitalopram concentration on [^11^C]UCB-J binding

We also investigated the effect of participants’ s-escitalopram level on [^11^C]UCB-J *V*_T_. The estimate of the effect of the log-transformed concentrations was +0.81 mL/cm^3^ per log[ng/L] (*r*_ρ_ = 0.18, *p* = 0.48) in the neocortex, and +0.39 mL/cm^3^ per log[ng/L] (*r*_ρ_ = 0.10, *p* = 0.70) in the hippocampus. The inclusion of age, sex, and IQ as covariates did not reveal any effect of s-escitalopram (Table S4).

### Exploratory analyses

#### Effects of escitalopram on hippocampus volume

The mean (SD) hippocampus volume was 4572 (389) mm^3^ in the escitalopram group versus 4767 (329) mm^3^ in the placebo group. When compared using a linear regression model controlling for age, sex, and intracranial volume the estimated difference was reduced to −97 mm^3^ (*p* = 0.33, Table S5). We further tested if there was an effect of escitalopram intervention duration, but a model including the *group-by-intervention duration* interaction term did not improve the model fit compared to a nested model, as compared using a likelihood-ratio test (*p* = 0.62). The effect of s-escitalopram concentration on hippocampus volume was estimated to be −22 mm^3^ per log[ng/L] of escitalopram (*p* = 0.34). All model estimates are listed in Table S5.

#### Effects of escitalopram on cortical thickness

Linear regression models with age and sex as covariates showed no difference in cortical thickness between the escitalopram group compared to the placebo group for the neocortical subregions (minimum *p* = 0.22, *p*_*adj*._ = 1.0). Estimates for individual regions are listed in Table S6. Likelihood-ratio tests between linear regression models including a *group-by-intervention duration* interaction term and nested models where the group term was excluded, did not support a time-dependent effect of escitalopram on cortical thickness in any of the subregions after correcting for multiple comparisons (minimum *p* = 0.033, *p*_*adj*._ = 0.16). Individual estimates are listed in Table S7. Lastly, s-escitalopram concentration also was not associated with cortical thickness (minimum *p* = 0.19, *p*_*adj*._ = 0.98) (Table S8).

## Discussion

In this study, we examine the effects of the SSRI escitalopram on brain synaptic density in SSRI-naïve healthy volunteers, as indexed by SV2A density measured with [^11^C]UCB-J PET. Administrating the drug to healthy participants allowed us to study potential effects on synaptic plasticity in the absence of clinical symptoms or brain pathology. The mean [^11^C]UCB-J *V*_T_s were not statistically significantly higher in the escitalopram group, and thus the group analysis did not support our primary hypothesis that [^11^C]UCB-J binding would be higher in the escitalopram group than the placebo group following 3–5 weeks of drug intervention. When adjusting for differences in the length of the intervention period within the escitalopram group, we found a time-dependent effect of escitalopram intervention on [^11^C]UCB-J *V*_T_, an effect that was more pronounced for the neocortex than the hippocampus. The time-dependent effect of escitalopram was reflected in the linear regression models estimating higher [^11^C]UCB-J *V*_T_ with increasing number of days of escitalopram intervention.

This positive association with escitalopram intervention duration suggests that a reason why we do not find a group difference in the primary analysis could be that an average of 28 days of escitalopram intervention is too short for synaptic effects to fully emerge. Delayed effects of the escitalopram intervention align with the clinical observations that when SSRIs are used for treating, e.g., depression, at least 2–4 weeks of treatment is required before effects on symptoms can be expected [29–31]. As our participants were healthy and relatively young and without cognitive impairments or a history of neuropsychiatric illness, it is also plausible that synaptic wiring, hippocampus volume, and cortical thickness, on which we saw no effect of escitalopram, are less affected by SSRIs. Effects sizes and temporal dynamics might be different in patients, as data from a recent [^11^C]UCB-J PET study by Holmes et al. [20] suggest that patients with depression have synaptic deficits that correlate with symptom severity. If replicated, it would be interesting to examine whether SSRI treatment normalizes SV2A levels and if such normalization is associated with clinical improvement.

The reason for the delay in symptom relief following the initiation of SSRI treatment is unclear, although both biological and neuropsychological hypotheses have been proposed, e.g., affective bias and reward sensitivity [11, 12, 27, 46]. Even though inhibition of the serotonin transporter occurs immediately after SSRI dosing [47], the net effect on synaptic serotonin levels is more dynamic. To the best of our knowledge, there is currently no in vivo method available for directly measuring serotonin levels in the human brain after weeks of SSRI intervention. A meta-analysis investigating the temporal effect of SSRIs on brain serotonin levels in rats found an initial dip in the frontal cortex followed by a linear increase over three weeks, in contrast to the hippocampus, where a marked increase was found on day 3 followed by a modest increase from day 6–21 [48]. Our data similarly estimate a larger average effect size for escitalopram in the hippocampus compared to the neocortex, but weaker association with intervention duration. The downstream effects of SSRIs on synaptic structures might be even further delayed and depend on the regional level of serotonergic innervation. One example of this was found in the rat hippocampus in response to the SSRI fluoxetine; in the subregion CA1, synaptic density was equally elevated following 5 and 14 days of intervention, whereas in the subregion CA3, the increase in synaptic density was significantly higher after 14 days than after 5 days of intervention [49] SSRI.

Aside from the intervention duration, the drug dose is also an important aspect to consider. Despite substantial variation in drug concentration, we saw no association between [^11^C]UCB-J *V*_T_ estimates and s-escitalopram concentration. This could be because we used a high daily dose of 20 mg escitalopram, which we expected to lead to a near-maximum occupancy of 70–80% of the serotonin transporter [47]. However, concentrations beyond the point of saturation of the serotonin transporter may be important for the engagement of low-affinity targets. Escitalopram is considered the most selective of the SSRIs [50], but could have important off-target effects according to a recent study: An allosteric binding site at the tropomyosin receptor kinase B (Trk-B) was identified as a low-affinity target of drugs representing several classes of antidepressants, including the SSRIs [51]. The Trk-B receptor activates neurotrophic signalling cascades when activated by brain-derived neurotrophic factor (BDNF). BDNF is known to have antidepressant effects and is increased in response to SSRIs, which forms a strong link between SSRIs and neuroplasticity [14]. It remains to be determined whether all SSRIs, including escitalopram, exert positive allosteric modulation of the Trk-B receptor at clinically relevant doses. This will be important for mapping out the mechanisms of SSRIs and could be a potential target for dual-action drugs promoting neuroplasticity. In this context, evaluating synaptic markers such as SV2A may prove to be a valuable tool.

Few other studies have investigated the effect of drug interventions on SV2A quantified with radioligand techniques. Using [^3^H]UCB-J in vitro autoradiography, we recently showed that a single administration of the 5-HT_2A_ receptor agonist psilocybin was associated with higher hippocampal SV2A levels in awake pigs 24 h after administration [52]. In contrast, another study found no effect of ketamine on SV2A binding in healthy individuals measured with [^11^C]UCB-J PET 24 h after the drug intervention [23].

So far, most other SV2A PET imaging studies have been cross-sectional case-control studies of neurodegenerative and psychiatric disorders for which causal relationships cannot be determined. Yet, indications of how modifiable SV2A is in the human brain may potentially be derived indirectly: A study on SV2A binding in cocaine-use disorder by Angarita et al. [53] found a negative correlation between [^11^C]UCB-J binding and duration of cocaine abstinence, whereas years of lifetime use was unrelated to SV2A binding. In contrast, another study found no association with the frequency of cannabis use in participants with cannabis use disorder [54]. Although exploratory, such analyses can indicate to which extent SV2A is a modifiable state marker or a stable trait marker of synapses.

Some methodological aspects of the current study should be considered. First, the use of SV2A as a proxy for pre-synaptic density. Although SV2A is ubiquitously expressed throughout the brain, it cannot be excluded that SSRI induced changes (or lack thereof) in SV2A binding estimates could have several different causes, such as a number of vesicles per synapse or differential effects on excitatory and inhibitory synapses. Preclinical studies comparing in vivo SV2A PET imaging with in vitro methods will help advance our understanding and interpretations of SV2A imaging studies.

Second, we chose *V*_T_ a priori as our primary outcome. The non-displaceable binding potential is often a preferred outcome for radioligands for which a reference region exist. However, the white matter, which has been proposed as a reference region for [^11^C]UCB-J, is known to contain some amounts of SV2A and overestimate the non-displaceable compartment [43, 44, 55]. Further, given that the centrum semiovale had the second highest estimated effect size (Cohen’s d of 0.51) and that we saw an effect of intervention duration for the centrum semiovale, we cannot exclude that the SSRI intervention could have an effect on the specific binding in the white matter, e.g. due to increased axonal transport of newly synthesized synaptic vesicle precursors [56]. The use of *V*_T_s also make our results more easily comparable to other [^11^C]UCB-J PET studies on related topics [20–23, 53, 57, 58].

Third, as our study did not include baseline [^11^C]UCB-J PET scans, we make an assumption of no group differences in cerebral SV2A binding before the intervention was initiated; this assumption is justified on the basis of balanced group randomization that took age and sex into account. The present study design also eliminates issues of long-term test-retest bias which has been reported to occur with [^11^C]UCB-J PET in some instances [59].

Finally, the sample size was targeted to detect larger effect sizes, which limits us in detecting subtler differences and subgroup differences (e.g., sex). As such, the study should be replicated in an independent sample, ideally with a longer range in the intervention period and in more subjects, to confirm the results and map the temporal dynamics more closely.

In summary, this is the first study to investigate the effect of an SSRI intervention, using clinically relevant doses and duration (i.e., 3–5 weeks), on pre-synaptic density in the human brain. Whereas we find no statistically significant group difference in SV2A, our secondary analyses suggest that escitalopram has a time-dependent effect on cerebral SV2A, i.e., that over 3–5 weeks, escitalopram induces synaptic neuroplasticity in the human brain. This offers a biological explanation for the delayed response commonly observed in patients treated with SSRIs. While replication of the findings is warranted, these results have important implications for future studies investigating the effects of SSRIs, especially concerning the duration of intervention studies. As such, our study adds a novel perspective to the growing literature on synaptic alterations in neuropsychiatric conditions.

### Supplementary information

Suplementary matrial

## Data Availability

Upon completion of the study, all data will be uploaded to the existing CIMBI Database [60]. Researchers may apply for access to the data.

## Code availability

The code generated and used in the production of this manuscript is available from the corresponding authors upon request.
