# Supplementary material for: Effects of escitalopram on synaptic density in the healthy human brain: a randomized controlled trial
Source: Mol Psychiatry. 2023 Oct 9;28(10):4272–9. doi: 10.1038/s41380-023-02285-8 (PMC10827655; doi:10.1038/s41380-023-02285-8)
Supplement: Supplementary file 1 — Suplementary matrial [file 41380_2023_2285_MOESM1_ESM.pdf]

Contents

IN / EXCLUSION CRITERIA 2
FIGURE S1: STUDY DESIGN 3
RADIOSYNTHESIS OF [^{11}C]UCB-J 3
RADIOMETABOLITE ANALYSIS 4
FREE FRACTION MEASUREMENT 4
1TCM V_T ESTIMATES FOR ADDITIONAL ROIS 6
Table S1 6
BP_{ND} ESTIMATES FROM THE SRTM2 IN PRIMARY ROIS 7
EFFECTS ON [^{11}C]UCB-J V_T 8
Table S2: Effect of intervention group 8
Table S3: Effect of intervention duration 9
Table S4: Effects of s-escitalopram 10
EFFECTS ON HIPPOCAMPUS VOLUME 11
Table S5:Effects of intervention group and s-escitalopram 11
EFFECTS ON CORTICAL THICKNESS 12
Table S6: Effects of intervention group 12
Table S7: Effects of intervention duration 13
Table S8: Effects of s-escitalopram 14

In / exclusion criteria

Inclusion criteria:

1. Healthy females and males aged 18-45 years.

Exclusion criteria:

1. Current or former primary psychiatric disorder (DSM-V or WHO ICD-11 diagnostic classification).
2. Current or previous neurological disease, severe somatic disease, or the consumption of drugs likely to influence the test results.
3. Current psychoactive medication.
4. Abnormal ECG.
5. Postural orthostatic tachycardia syndrome.
6. Hypotension (blood pressure < 100/70 mmHg) or hypertension (blood pressure > 140/90 mmHg).
7. Head injury or concussion resulting in loss of consciousness for more than 2 min.
8. Pregnancy.
9. Lactation.
10. Alcohol or drug abuse.
11. Nicotine addiction.
12. Recreational drug use other than tobacco and alcohol within the last 30 days.
13. Cannabis use > 50 x lifetime.
14. Recreational drug use > 10 x lifetime (for each substance).
15. Non-fluent in Danish or pronounced visual or auditory impairments.
16. Current or past learning disability.
17. Severe physical impairments affecting eyesight or motor performance.
18. Participation in experiments with radioactivity (>10 mSv) within the last year or other significant exposure to radioactivity.
19. Contraindications for magnetic resonance imaging (MRI).
20. Allergy to compounds used for PET scan or intervention.

Figure S1: Study design

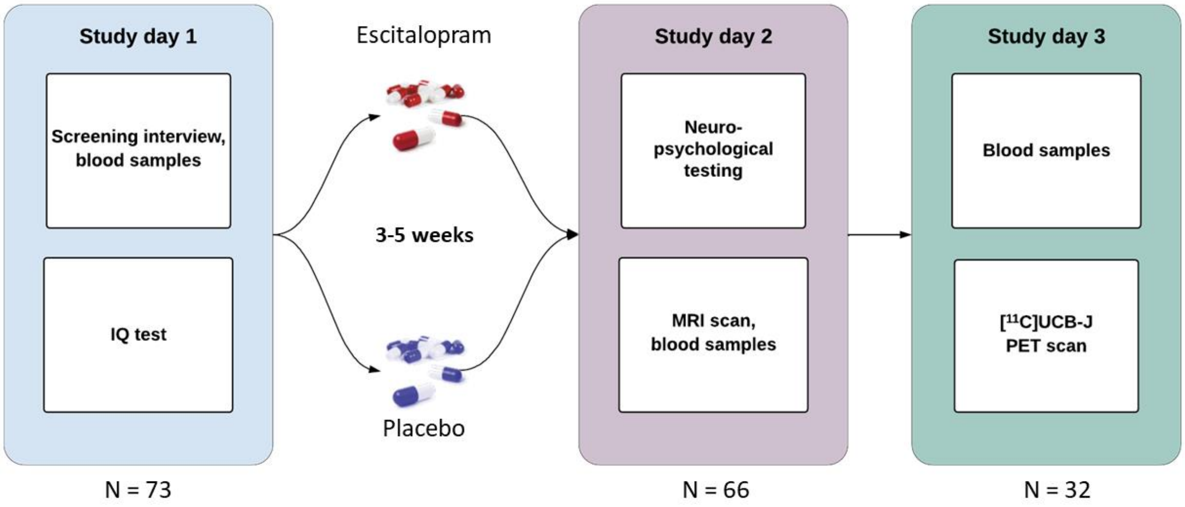

Figure S1: Study design. Participants were screened and randomized on Study day 1. Within 1-5 days, participants started the intervention with either escitalopram or placebo capsules. After 3-5 weeks of full dose intervention, participants came back for MRI scan and neuropsychological testing on Study day 2 and PET scan on Study day 3.

Radiosynthesis of [^{11}C]UCB-J

Proton irradiation of the target material (nitrogen-14 gas) was performed using the cyclotron: Scanditronix MC-32 with aluminium high-pressure gas target. Irradiations for carbon-11 were performed at 16 MeV. The target gas used was 10% hydrogen in nitrogen. Following irradiation, the target gas was transferred to the radiochemistry system (Scansys Aps) through stainless steel capillaries. [^{11}C]methyl iodide was synthesized from [^{11}C]methane by a standard circulation procedure.

Preparation of the precursor: The precursor (1.5 mg) was dissolved in MeOH (70 µL) and 1 M HCl (15 µL) was added. The resulting mixture reacted overnight at room temperature. On the day of synthesis, the liquid was removed by a stream of nitrogen to complete dryness.

Preparation of the labelling mixture: 4-5 mg P(o-tolyl)$_3$ was dissolved in DMF (1.8 mL) and H_2O (0.2 mL) in a capped vial and degassed with nitrogen. Pd_2dba_3 (4-5 mg) was weighed out in a capped vial and flushed with nitrogen. Immediately before labelling, the P-(o-tolyl)$_3$ solution was added to the Pd_2dba_3. From here, 350 µL was withdrawn and added to a 0.9 mL vial containing 0.5 M K_2CO_3 (20 µL). The formed [^{11}C]methyl iodide was trapped in the 0.9 mL glass vial containing the K_2CO_3/ P(o-tolyl)$_3$/Pd_2dba_3 mixture. After trapping the [^{11}C]methyl iodide, the hydrolyzed DM-BF$_3$-UCB-J precursor was re-dissolved in DMF (150 uL) and reacted with the mixture by heating at 100 °C for 300 seconds to give [^{11}C]UCB-J. The reaction mixture was diluted

with 4 mL 0.1% H3PO4 and automatically injected onto a preparative HPLC-column (Onyx™ Monolithic C-18, 100 × 10 mm equipped with a SecurityGuard Cartridge Lux Cellulose-4, 4 x 3.0 mm; flow: 6 mL/min; eluent: 13/87 [ethanol (96%)/0.1 M phosphoric acid]). The radioactive fraction corresponding to the radiolabeled product (retention time (rt) app. 300 s) was collected by diverting the flow from the column outlet through a 0.22 µm sterile filter and directly into a sterile stoppered and a capped vial containing phosphate buffer (9 mL, pH 7). The identity and molar activity of the product was determined by using a C-18 column (Kinetex 2.6 um, C18, 100Å, 50 x 4.6 mm, Phenomenex) eluted with 67% 25 mM citrate buffer (pH 5.4)/33% acetonitrile ; injection volume 50 µL; flow rate 1.5 mL/min; on-line UV (261 nm) and radioactivity detection. Rt UCB-J 2.1 min.

Radiometabolite analysis

For measurement of intact tracer and radiolabeled metabolites, plasma samples up until 90 min were filtered through a 0.45 µm syringe filter (Whatman GD/X 13 mm, Cytiva, Buckinghamshire, UK) and subsequently diluted 1:1 with 20 mM disodium hydrogen phosphate and 5 mM sodium-1-decanesulfonate pH 7.2 with 2% isopropanol. Samples were analyzed in a fully automated column-switching HPLC system (UltiMate 3000, Thermo Fisher Scientific, Hvidovre, Denmark) connected to a radio-HPLC detector (PosiRam Model 2, LabLogic Systems, Sheffield, UK)(Gillings, 2009). The HPLC system was equipped with a small extraction column (Shimpack MAYI-ODS 30x4.6 mm, Shimadzu, Ballerup, Denmark) combined with an analytical column (Onyx Monolithic C18 50x4.6 mm, Phenomenex, Brønshøj, Denmark). For extraction and elution, the mobile phase consisted of 100% 20 mM disodium hydrogen phosphate and 5 mM sodium-1-decanesulfonate pH 7.2 with 2% isopropanol and 59% 100 mM sodium dihydrogen phosphate and 2 mM sodium-1-decanesulfonate pH 2.6 and 41% methanol, respectively. Samples were injected in a volume of 4 mL, and the analysis was run at a flow of 5 mL/min at 25 °C. The total runtime for each sample was 8.55 min with a 4 min extraction step, 4 min elution step and 0.55 min of equilibration. Four eluate fractions were collected in 2 min intervals, and radioactivity was subsequently measured using a gamma counter (Wizard 2480, Perkin Elmer, Wallac Oy, Turku, Finland). The parent tracer fraction was calculated as follows: % parent fraction = (radioactivity of parent eluate/total amount of collected radioactivity) x 100%.

Free fraction measurement

[^{11}C]UCB-J free fraction was measured in the following way: plasma spiked with 1 µL/mL of tracer was added into one dialysis chamber (500 µL chambers, Harvard Apparatus, Holliston, MA, USA) and dialyzed against an equal volume of phosphate-buffered saline through a cellulose membrane (MWCO 10,000 Dalton, Harvard

Apparatus, Holliston, USA). The system was incubated at 37 ºC for 30, 60, 120, 150 and 180 min. After completion of dialysis, samples were extracted and analyzed for radioactivity using the Cobra II gamma counter (Packard Instrument Company, Meriden, USA). The ratio of buffer:plasma radioactivity was plotted over time and fitted according to eq. 1 with a fixed average value of the diffusion coefficient (k_D), using GraphPad Prism (v. 9.0, GraphPad Software, San Diego, CA, USA) to determine f_{Peq}.

$$f_P(t) = \frac{f_{Peq} \times t}{k_D + t} \qquad \text{(eq. 1)}$$

[^{11}C]UCB-J plasma free fraction did not differ between intervention groups or sexes, but correlated with age (β_{esc} = 0.01, p = 0.52; β_{male} = -0.003, p = 0.85; β_{age} = -0.005 /year, p = 0.005). The effect of age was slightly reduced and not statistically significant when omitting one age-outlier of 41.9 years (β_{age} = -0.004, p = 0.1). As there was no group difference nor association between [^{11}C]UCB-J V_T estimates and f_P in the neocortex (βf_P = 7.0 mL/cm^3, p = 0.50), it was not used in the analyses.

1TCM V_T estimates for additional ROIs

Table S1

Region	Placebo (N=12)	Escitalopram (N=17)	p	Cohen's *d*
**Neocortex**	17.55 (1.96)	18.25 (2.51)	0.41	0.31
**Hippocampus**	14.25 (1.85)	15.12 (2.21)	0.26	0.43
**Caudate**	20.23 (2.31)	21.83 (3.63)	0.16	0.53
**Centrum semiovale**	3.85 (0.53)	4.14 (0.61)	0.18	0.51
**Insula**	18.21 (2.05)	19.38 (2.64)	0.19	0.50
**Putamen**	20.52 (2.25)	21.77 (3.02)	0.21	0.47
**Orbitofrontal ctx**	17.78 (2.16)	18.85 (2.94)	0.27	0.41
**Entorhinal ctx**	14.05 (2.70)	15.19 (2.82)	0.28	0.41
**Med inf temp gyrus**	17.97 (1.93)	18.90 (2.70)	0.29	0.39
**Amygdala**	16.66 (2.18)	17.60 (2.75)	0.31	0.38
**ACC**	18.67 (2.28)	19.65 (2.94)	0.32	0.37
**Sup frontal gyrus**	16.96 (2.08)	17.81 (2.61)	0.34	0.36
**vlPFC**	17.95 (2.07)	18.83 (2.75)	0.34	0.36
**dlPFC**	17.69 (2.14)	18.49 (2.59)	0.37	0.34
**Sensory-motor ctx**	16.21 (2.02)	16.93 (2.30)	0.38	0.33
**Temporal ctx**	18.36 (1.97)	19.12 (2.60)	0.38	0.33
**Parietal ctx**	17.77 (2.09)	18.42 (2.53)	0.45	0.28
**Cerebellum (excl. vermis)**	13.93 (1.57)	14.41 (1.93)	0.47	0.27
**Thalamus**	17.35 (2.15)	17.91 (2.41)	0.51	0.25
**Sup temp gyrus**	18.84 (2.04)	19.42 (2.50)	0.50	0.25
**Raphe nuclei**	10.98 (1.52)	11.13 (1.25)	0.78	0.11
**Occipital ctx**	17.50 (1.94)	17.67 (2.39)	0.84	0.08
**PCC**	18.26 (1.81)	18.40 (2.33)	0.86	0.07

BP_{ND} estimates from the SRTM2 in primary ROIs

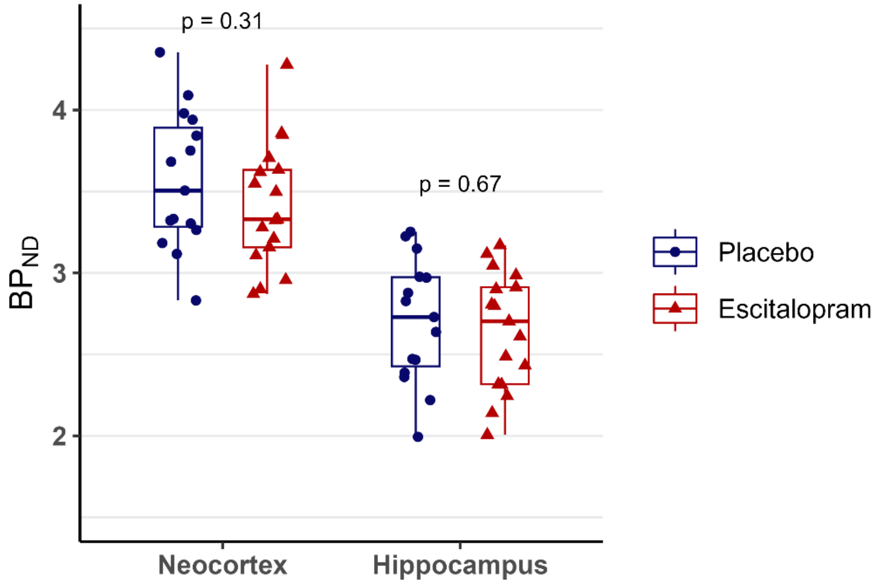

Effects on [^{11}C]UCB-J V_T

Table S2: Effect of intervention group

General linear model with age, sex and IQ as covariates to test group effect on [^{11}C]UCB-J V_T

	**Neocortex**			**Hippocampus**			**Centrum semiovale**		
Predictors	*Estimate*	*SE*	*p*	*Estimate*	*SE*	*p*	*Estimate*	*SE*	*p*
Intercept*	17.01	0.92	**<0.001**	13.75	0.82	**<0.001**	3.83	0.25	**<0.001**
Age (per decade)	0.23	0.84	0.79	0.14	0.76	0.86	0.01	0.23	0.98
Sex (Male)	-1.48	0.85	0.09	-1.29	0.76	0.10	-0.22	0.23	0.35
IQ	0.14	0.06	**0.027**	0.13	0.05	**0.026**	0.02	0.02	0.37
Intervention (escitalopram)	-0.11	0.88	0.90	0.16	0.79	0.84	0.19	0.24	0.43

* Intercept centered at age = 20 years and IQ = 100.

Table S3: Effect of intervention duration

General linear model with age, sex and IQ as covariates to test effect of intervention duration on [^{11}C]UCB-J V_T

	Neocortex				Hippocampus				Centrum semiovale			
Predictors	*Estimate*	*SE*	*p*	r_p	*Estimate*	*SE*	*p*	r_p	*Estimate*	*SE*	*p*	r_p
Intercept*	17.18	0.78	**<0.001**		13.85	0.78	**<0.001**		3.86	0.24	**<0.001**	
Age (per decade)	-0.32	0.74	0.67	-0.09	-0.23	0.74	0.76	-0.07	-0.12	0.22	0.61	-0.11
Sex (Male)	-1.65	0.82	0.056		-1.22	0.82	0.15		-0.21	0.25	0.41	
IQ	0.15	0.05	**0.008**	0.53	0.13	0.05	**0.017**	0.48	0.02	0.02	0.29	0.22
Intervention (escitalopram)	0.32	0.76	0.68		0.42	0.76	0.59		0.28	0.23	0.23	
Duration (placebo)	-0.01	0.13	0.95	-0.01	-0.06	0.13	0.62	-0.1	-0.02	0.04	0.60	-0.11
Duration (escitalopram)	0.47	0.14	**0.003**	0.58	0.30	0.14	**0.048**	0.41	0.10	0.04	**0.029**	0.45

*Intercept centered at age = 20 years, mean intervention duration (29 days), and IQ = 100. R_p = partial correlation coefficient.

Table S4: Effects of s-escitalopram

General linear model with age and sex as covariates to test effect of s-escitalopram level on [^{11}C]UCB-J V_T

	Neocortex			Hippocampus			Centrum semiovale		
Predictors	*Estimate*	*SE*	*p*	*Estimate*	*SE*	*p*	*Estimate*	*SE*	*p*
Intercept*	17.03	0.92	**<0.001**	13.80	0.83	**<0.001**	3.85	0.26	**<0.001**
Age (per decade)	0.23	0.83	0.78	0.17	0.75	0.83	0.03	0.23	0.91
Sex (Male)	-1.50	0.86	0.01	-1.31	0.78	0.11	-0.22	0.24	0.36
IQ	0.14	0.06	**0.028**	0.13	0.06	**0.026**	0.02	0.02	0.37
S-escitalopram (log[ng/L])	-0.04	0.20	0.85	0.01	0.18	0.96	0.03	0.06	0.57

* Intercept centered at age = 20 years.

Effects on hippocampus volume

Table S5:Effects of intervention group and s-escitalopram

General linear model with age, sex and intra-cranial volume (ICV) as covariates to test effect of intervention group and s-escitalopram level on hippocampus volume

	Group			S-escitalopram		
Predictors	*Estimate*	*SE*	*p*	*Estimate*	*SE*	*p*
Intercept*	4637	99	**<0.001**	4736	91	**<0.001**
Age (per decade)	68	104	0.52	61	103	0.56
Sex (Male)	-124	117	0.23	-128	117	0.28
ICV (L)	2048	419	**<0.001**	2038	423	**<0.001**
Intervention (Escitalopram)	97	99	0.33			
S-escitalopram (log[ng/L])				-22	23	0.34

Hippocampus volume is in unit of mm^3. * Intercept centered at age = 20 years, and mean ICV.

Effects on cortical thickness

Table S6: Effects of intervention group

General linear model with age and sex as covariates to test effect of intervention group cortical thickness

	Frontal			Parietal			Temporal			Occipital			Insular		
Predictors	*Estimate*	*SE*	*p*	*Estimate*	*SE*	*p*	*Estimate*	*SE*	*p*	*Estimate*	*SE*	*p*	*Estimate*	*SE*	*p*
Intercept*	2.77	0.02	**<0.01**	2.52	0.03	**<0.01**	2.90	0.03	**<0.01**	2.02	0.03	**<0.01**	3.16	0.04	**<0.01**
Age (per decade)	-0.07	0.03	**0.02**	-0.05	0.03	0.13	-0.04	0.04	0.32	-0.01	0.03	0.67	-0.05	0.05	0.29
Sex (Male)	0.06	0.03	**0.04**	0.06	0.03	0.07	0.08	0.04	0.06	0.06	0.03	0.07	0.06	0.04	0.17
Intervention (Escitalopram)	0.03	0.03	0.22	0.03	0.03	0.26	0.01	0.04	0.77	0.01	0.03	0.83	-0.00	0.04	0.92

Cortical thickness is in the unit of mm. * Intercept centered at age = 20 years. All escitalopram corrected p-values = 1 (Bonferroni-Holm, 5 tests).

Table S7: Effects of intervention duration

Likelihood-ratio tests of model with *group-by-intervention duration* vs. a nested model without group term. Age and sex included in both models

Frontal			Parietal			Temporal			Occipital			Insular		
χ^2	p	$p_{adj.}$	χ^2	p	$p_{adj.}$	χ^2	p	$p_{adj.}$	χ^2	p	$p_{adj.}$	χ^2	p	$p_{adj.}$
6.72	0.03	0.16	3.1	0.21	0.62	1.60	0.45	0.90	6.84	0.03	0.16	1.00	0.61	0.90

($p_{adj.}$ = Bonferroni-Holm, 5 tests).

Table S8: Effects of s-escitalopram

General linear model with age and sex as covariates to test effect of s-escitalopram on cortical thickness

	Frontal			Parietal			Temporal			Occipital			Insular		
Predictors	*Estimate*	*SE*	*p*	*Estimate*	*SE*	*p*	*Estimate*	*SE*	*p*	*Estimate*	*SE*	*p*	*Estimate*	*SE*	*p*
Intercept*	2.77	0.02	**<0.01**	2.52	0.03	**<0.01**	2.90	0.04	**<0.01**	2.02	0.03	**<0.01**	3.16	0.04	**<0.01**
Age (per decade)	-0.07	0.03	**0.02**	-0.05	0.03	0.15	-0.04	0.04	0.34	-0.01	0.03	0.67	-0.05	0.04	0.28
Sex (Male)	0.06	0.03	**0.03**	0.06	0.03	0.07	0.08	0.04	0.06	0.06	0.03	0.07	0.06	0.04	0.18
S-escitalopram (log[ng/L])	0.01	0.01	0.19	0.01	0.01	0.28	0.00	0.01	0.88	0.00	0.01	0.79	-0.00	0.01	0.97

Cortical thickness is in the unit of mm. * Intercept centered at age = 20 years. All escitalopram corrected p-values = 1 (Bonferroni-Holm, 5 tests).
